# Impact of COVID-19 on distance learning practical design courses

**DOI:** 10.1007/s10798-023-09806-0

**Published:** 2023-01-21

**Authors:** Lina Nageb Fewella

**Affiliations:** Department of Interior Design and Furniture, Faculty of Applied Arts, Damietta University, Corniche El-Nile St, El-Usar, Damietta Main, Damietta, Postal No 34511 Egypt

**Keywords:** COVID-19 pandemic, e-Learning, Art education, Applied arts, Design education, Developing countries

## Abstract

This paper explores the impact of COVID-19 on higher education practical design courses in Egypt. Because of inadequate resources and preparedness, Egyptian colleges have struggled to adopt digital teaching methods during the COVID-19 pandemic. This paper examines strategies that are the most feasible for teaching practical courses during or after a pandemic through distance learning (on online platforms). An action research project was set up to deliver two studio-based design courses, one on architectural drawing and the other on furniture design via distance learning (online mode). This approach used a suite of technologies and synchronous and asynchronous delivery mechanisms, such as Zoom and Google Classroom. Student perceptions about the impact of these changes were evaluated using questionnaires. A psychological effect of the conditions caused by the pandemic on students has been the loss of interest in academics. The research results partially support the use of online platforms to teach practical courses. However, more needs to be done to improve the delivery of online courses in Egypt. Further, holding competitions was found to boost students’ motivation levels. A future strategy for teaching practical courses in applied arts and engineering is proposed in this paper.

## Introduction

Hammad (2018) notes that humans learn differently based on variations in culture, priorities, tools, and other factors. With the onset of the pandemic, social distancing became a disease control pillar because COVID-19 spreads through respiratory droplets (Pitout & Finn, 2020). Thus, the pandemic has transformed education by causing learning at home to become the norm (Keswani et al., 2020). In this situation, the value of comssmunications technology increased exponentially, allowing individuals to work and learn from home (Király et al., 2020). Technology’s influence on education has revolutionized learners’ actions and behaviors and transformed how they study and interact. Hence, teachers have reshaped learning processes and modified learning materials to reflect this transition (Okaz, 2015). The transition to online training has caused college lecturers to struggle because of insufficient time or expertise to handle their work (Plummer et al., 2021). Megahed et al. (2019) claim that technology has infiltrated and integrated into every aspect of society, enabling faster and simpler access to information, greater flexibility, and more affordability. Students are interested in innovations and technologies for their immense potential in education. Students have also embraced “self-learning” via e-learning-based classroom instruction, which functions as a bridge between instructors and students (Phutela & Dwivedi, 2020).

This study explored the impact of COVID-19 on higher education in universities, particularly the delivery of practical courses online. A practical course is an activity-based education strategy that includes learning via hands-on projects and building teamwork skills (Pawar et al., 2020). We applied the study to first-year students of the Department of Interior Design and Furniture, Faculty of Applied Arts. This paper aimed to determine the most effective approaches for distance learning online design studio courses during and after the pandemic.

## Literature review

### Implications of the spread of COVID-19 on higher education

On 11 March 2020, the World Health Organization declared the COVID-19 outbreak a pandemic (Király et al., 2020). The difficulties of combating the COVID-19 pandemic have halted or slowed many educational facilities and economic and socio-cultural activities (Haghani et al., 2020). These circumstances led staff and companies to implement work-from-home policies relying on online platforms instead of the in-office work arrangement to observe social distancing (Király et al., 2020). The internet has played a paramount role in promoting e-learning during the pandemic (Favale et al., 2020). Academics re-evaluated how technology could be utilized at all educational levels to continue knowledge dissemination. Studying the efficacy and students’ perspectives regarding introducing technology into education is crucial, particularly in areas where e-learning is rare (Wijenayaka & Iqbal, 2021). E-learning refers to remote education for students outside the conventional classroom. Instructors must track student progress and ask questions to ensure understanding (Singh et al., 2020). Students may learn via social media and webinars (Keswani et al., 2020). Moodle, Edmodo, Desire2Learn, Google Classroom, and other learning management systems (LMSs) are mediums that have been used to deliver online lectures (Kumar et al., 2020). However, courses like practical chemistry are seldom taught online. Wijenayaka and Iqbal (2021) have shown that the undergraduate chemistry curriculum must include laboratory work to foster practical chemical skills. Because of “fast-changing” social conditions and the pandemic-related social distancing- policy, laboratory-based sessions became difficult to conduct. Therefore, academic and higher education institutions must use technology-based solutions while developing practical chemistry curriculums.

### Nature of practical design courses before COVID-19

Each science discipline has distinctive traits, prerequisites, and teaching philosophies (Ceylan et al., 2021). Learning a studio-based art curriculum using digital tools can be challenging. Learning technology may seem incongruous with the creative product development of artists who do not utilize digital production tools because artistic creativity is studio-based design courses (Koh & Kan, 2020). Students use interactive studios for leisure and to produce design work, engage, and discuss with each other (Ceylan et al., 2021). Project-based learning emphasizes social and collaborative skills, providing instruction and experience to students. Thus, students’ problem-solving, workplace creativity, and planning is enhanced (Pawar et al., 2020; Sakulviriyakitkul et al., 2020). Applied Arts combines art and engineering education. Fewella et al., (2021) suggest that engineering drawing courses should be taught using recorded videos and online projects that can be used in practical workshops. Few students are present in design classes that require professor–student interaction and evaluation. As such the few students present in such classes due to capacity issues that online classes can help reduce. Alternatives, such as the Zoom videoconferencing platform, that facilitate interactive conversations and drawing adjustments, should be used. The studio space is used to host seminars, lectures, juries, one-on-one critiques, and collaborative or individual work and thus promotes people’s interaction. In the twenty-first century, design studios have various options. Technological innovations have the potential to replace traditional communication and representation methods, thus making way for blended learning and virtual and online studios (Ceylan et al., 2021).

### Nature of practical design courses during COVID-19

Art and design education has developed new approaches in response to societal changes and global issues (Kokko, 2021). Google Classroom was the most popular online education platform during the COVID-19 pandemic because of its ease of use, whereas Moodle was the most popular platform before the pandemic (Setiawan et al., 2021). Google Classroom is an affordable, accessible, and user-friendly LMS. It offers blended and flipped learning modes, an open-source smartphone application, simplicity, timesaving features, flexibility, cloud support, and digital learning (Kumar et al., 2020). Course content is critical for the success of online learning (Chatterjee et al., 2020; Singh et al., 2020). The school system should employ ICT resources (Gubiani et al., 2019). Students undertaking practical engineering courses must be aware of drawing tools such as AutoCAD. E-learning sites offer videos and drawings for each subject (Khoroshko et al., 2019; Vogt, 2018).

The flipped classroom model presents external technical material through a virtual platform, creating a friendly atmosphere in the classroom that facilitates dialogue, problem-solving, and active learning (Bhat et al., 2020). Contrary to some perceptions, a hybrid strategy with digital tools improves course results (Vivek & Ramkumar, 2021). Ceylan et al. (2021) evaluated an online design studio active during the pandemic. Students valued the studio’s digital tools. The researchers found that with the correct tools and encouragement, distance education can be effective for students’ learning. Alhusban et al. (2022) indicated that most architects believe COVID-19 would impact architectural design, future work, learning, leisure, and teaching environments. Future design should use renewable, power- and water-independent energy. House designs should emphasize interior design, transparency, private areas, quality of life, natural lighting and ventilation, good indoor air quality, use of plants and natural materials, and indoor–outdoor linkages. Interior and architectural design curriculums should be updated accordingly.

### Advantages of e-learning during the COVID-19 pandemic

Instructors have conceived innovative ways of creating new educational content; they hold global video conferences for students using software like Zoom. These instructors often have no previous experience dealing with e-learning materials (Yuen & Xie, 2020). Megahed et al. (2019) have confirmed that technology offers advantages, such as quicker and easier access to information, greater flexibility, and affordability. Students have become rapidly interested in emerging innovations and technologies because there is tremendous scope for employing technological innovations in the education sector (Violante & Vezzetti, 2014). Students engage in “self-learning” through e-learning classrooms, and teaching is an essential bridge in teacher–student interactions (Phutela & Dwivedi, 2020). Many have recognized that videoconferencing is not inferior to face-to-face instruction; this will help future generations of students in the post-pandemic context (Yuen & Xie, 2020).

Students are familiar with technology integrated into education that produces social skills. Cooperation, commitment, conversation, thinking, problem-solving techniques, and metacognitive skills are the social abilities that are essential for today’s students to adapt suitably to the novel situation of e-learning (Tzifopoulos, 2020). Digital platforms address several problems, such as eliminating old, ineffective teaching methods and the shortage of teachers for specific topics, and foster effective e-learning environments (Phutela & Dwivedi, 2020). E-learning is now adopted frequently because of the multiple advantages it presents (Phutela & Dwivedi, 2020; Purg et al., 2018). Students in engineering faculties have an aptitude for innovation and are interested in digital education (Bhat et al., 2020); hence, they interact with e-learning more positively than others. Kumar et al. (2020) reported on higher education students’ experience of using Google Classroom in Malaysia. They found that the platform was easy to access even for those with no previous experience with online platforms. Furthermore, it easily integrates with other applications, such as YouTube, and is linked to Google Drive’s cloud storage.

Blended learning became beneficial and popular among educators and learners during the pandemic. It made online education convenient and flexible, gave students access to additional resources, and helped students develop critical thinking skills. El-Sofany and El-Haggar (2020) note that e-learning has several essential outcomes, including enhancing student capabilities in using mobile technology for learning, positive academic awareness, and flexible access to mobile services for educational content. Nonetheless, there were several challenges in e-learning. Teachers and students are exhausted from constantly learning and adapting to new technology and completing extra responsibilities and duties, among other issues (Yang, 2021).

### COVID-19 e-learning challenges and solutions

Differences in the skill level, cultural background, ethnicity, and personality of students make university education complicated (Okaz, 2015). Gubiani et al. (2019) assert that e-learning should lead to modifications in traditional approaches to encourage student engagement. Professors need training in blended learning just as e-learners must have computer and internet skills. Interactive content development requires technology and skills. Proper training and rapid assistance should be offered. Creating new materials, responding to student feedback swiftly (through forums or e-mails), and updating online courses require much effort. Professors should use high-quality open educational resources to save time for other tasks. Elhaty et al.’s (2020) study on the adoption of e-learning for university practical courses despite the COVID-19 crisis showed that science students and educators worry more about inadequate practical skills than social science students. Notably, 55.6% teachers and 40.8% students preferred live teaching and recording of practical courses. However, Phutela and Dwivedi (2020) noted that in e-learning, students are interactive. Nonetheless, they also observe that face-to-face interaction, which is low in e-learning, is essential for education, and that learning is more manageable during face-to-face communication (Violante & Vezzetti, 2014). Kumar et al. (2020) posit that Google Classroom’s user interface is tiresome and that internet connectivity issues make private messaging between students problematic. Users pointed to the need for improving the interface and navigation and increasing privacy. Others have advised utilizing the application offline or enhancing the university internet connection facility. The pandemic has affected the functioning of the education system; however, it has presented an opportunity to develop and implement alternative educational approaches (Keswani et al., 2020). Gaida et al. (2016) and Zhu and Xu (2021) note that soft skills in studio education should be developed to enable students to share designer viewpoints and oral expressive abilities. Yuen & Xie (2020) recommended three points teachers should follow during and after a pandemic, including providing students with realistic simulation technology. Habib et al. (2021) identified modern technology, an interactive classroom, internet use, and online content as essential e-learning factors. Problem-solving and experience-gathering should be emphasized in art education, while recognizing current industrial needs and retaining studio students to serve the community, integrate, and evolve (Zhu & Xu, 2021).

According to Asadpour’s (2021) study, lockdowns and stay-at-home orders resulting from the COVID-19 outbreak challenged architectural design courses, exposing the shortcomings of traditional education systems and presenting the opportunity for reform. Design studio students rejected self-directed learning and wanted tutors to do more. In developing countries, the education system focuses on teaching skills and neglect motivating and encouraging students. Since e-learning prioritizes individual abilities, sustainable architectural education is possible. Short-term seminars and workshops may combine old and new ideas to show the current path and restore normality. New configurations should rethink e-studio contents, procedures, and outcomes to improve student learning, assessment performance, and course goals. E-design studios need emotional, social, and economic support. Students and teachers should anticipate the drawbacks of social media. Restructuring and utilizing national and university resources is also necessary. E-learning is also affected by wealth inequality. Internet connectivity at architecture schools must improve. Alsuwaida (2022) recommended that professors must organize and deliver online art and design courses appropriately to improve students’ understanding and assimilation. Some instructors overburden learners, causing confusion. Teachers should set clear objectives and guide students to reach them so that they realize how difficult things become easier with repetition and persistence. Instructors should be trained to integrate technology into higher education pedagogy to promote technological literacy.

## Methodology

The COVID-19 pandemic hit hard during the second semester of the 2019–2020 academic year. This research looks at how traditional approaches to education in the Applied Arts can be altered, in whole or in part, to accommodate students’ needs during the epidemic. We selected two courses following a practical approach from the Department of Interior Design and Furniture: Furniture Design and Architectural Drawing for first-year students. In the faculty studios before the epidemic, these courses were traditionally taught using manual drawing sheets as part of a project-based learning curriculum. When the pandemic hit, a more convenient teaching method became necessary: distance learning. The students were in their first year and had not been previously exposed to engineering design programs; this was our biggest challenge. Because of this, the use of technology in the classroom was restricted to electronic platforms and the use of engineering software by university lecturers as an explanatory aid. At the beginning of the pandemic, students were taught exclusively via online courses; afterward, the Ministry of Higher Education directed that colleges use a hybrid education model.

This study focuses on the initial phase of remote learning to establish the details of a technology-based teaching approach during a pandemic. Except for Engineering Perspective for first-year students, e-learning is not used for any courses offered by the department. Before the outbreak of the pandemic in 2019, blended learning was practiced separately as an expermental approach. All departments studied the same Engineering Perspective drawing course taught using YouTube videos in Google Classroom. Students took the course online and in the classroom, where they learned how to navigate Google Classroom and access course materials. Nevertheless, design drawings and discussions about them were not corrected or debated online (Fewella et al., 2021).

**Fig. 1:**
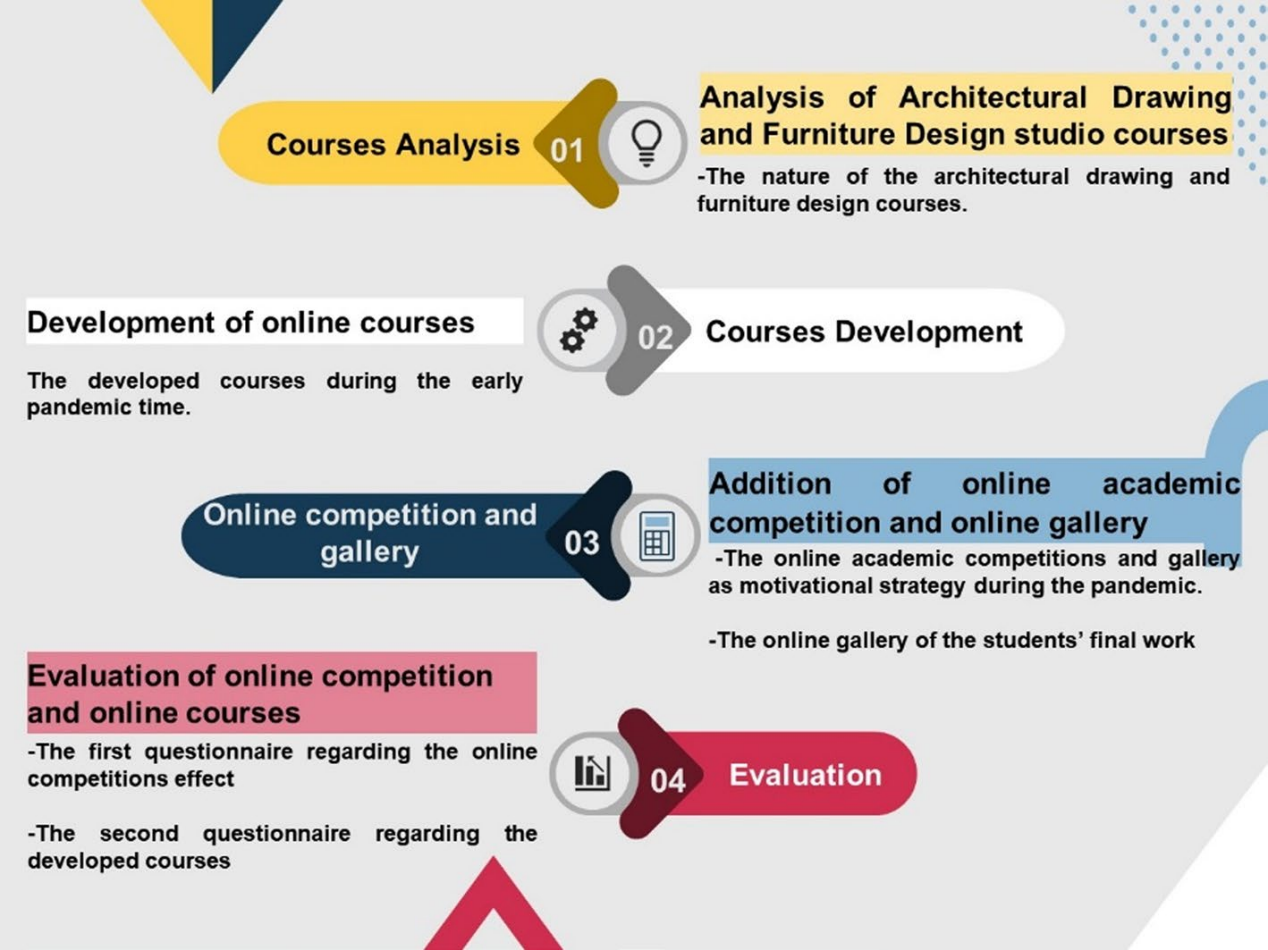
Course development approach and results in the evaluation. The template design application used is PresentationGO

This study presents a comprehensive online experiment grounded in this prior preparation for e-learning by students throughout the pandemic. Adopting traditional teaching approaches for e-learning required extra time and efforts from the instructor and students when a new online education system was introduced. Further, the first-year students had not been exposed to drawing and design software like AutoCAD and 3Ds Max. These two courses, which relied heavily on drawings and face-to-face contact as part of their project-based learning framework, presented a significant challenge. The study's outcome presents a method for gradually transitioning the mode of classes from their present, more traditional format to an online one while maintaining students’ interest and engagement. Additionally, we designed a survey to discover what students thought of the new approach and how they might improve it. Figure 1 presents the research strategy of the developmental approach of the course.

### Settings and participants

**Fig. 2:**
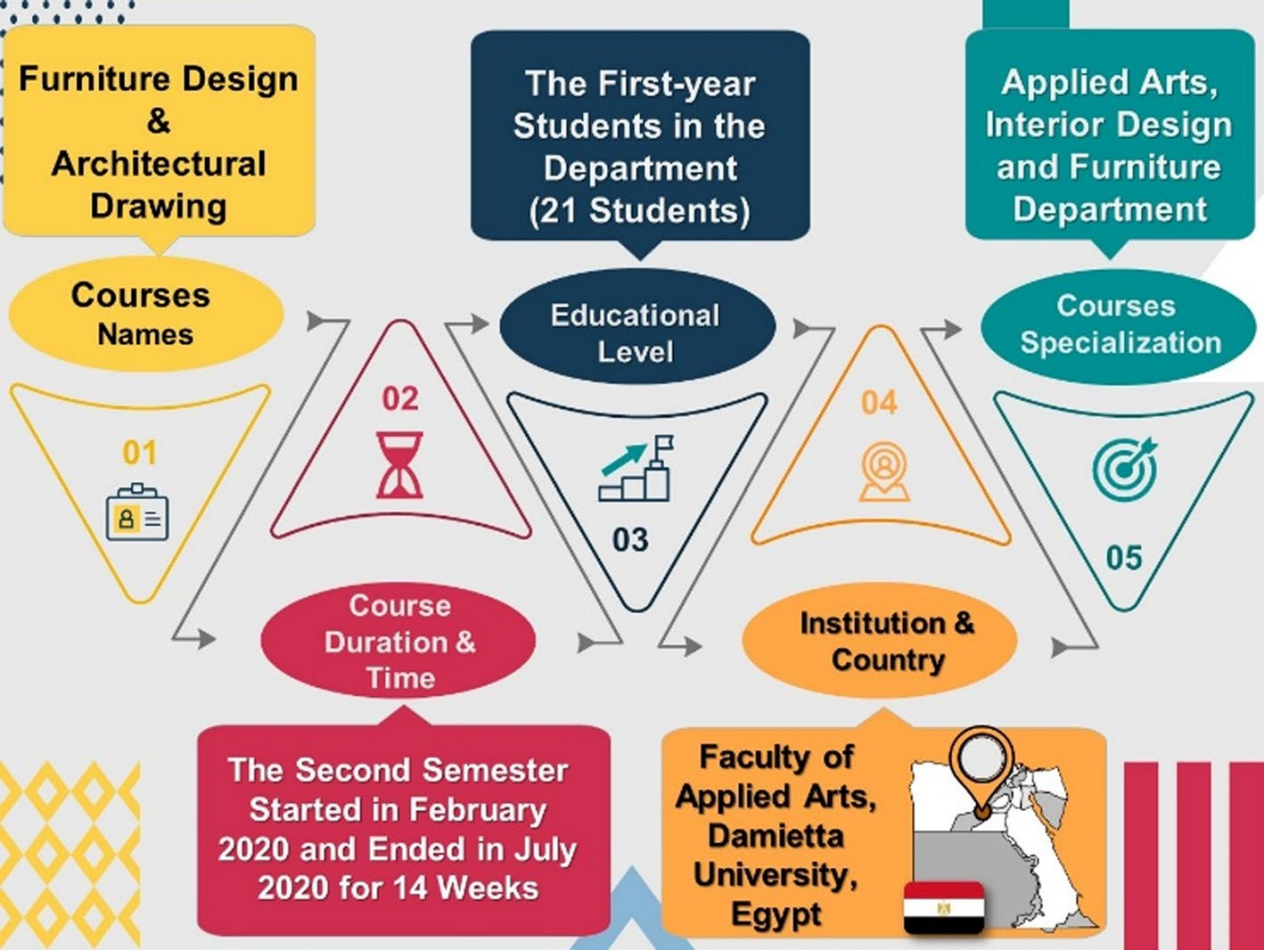
The courses’ settings and participants. The template design application used is PresentationGO

The present research focuses on two courses of the Department of Interior Design and Furniture at the Faculty of Applied Arts in Egypt. The chosen courses were practical design courses conducted in the design hall studio. The two courses were Furniture Design and Architectural Drawing, which came under engineering drawing. These courses were considered because they are first-year courses. Considering the requirement of physical distance and large-sized drawing sheets and tables, the number of students who were admitted was limited. First-year Interior Design students in 2019–2020 numbered 21. Figure 2 presents the courses’ settings and participants.

### Analysis of Architectural Drawing and Furniture Design Studio Courses

These courses were held in the design studios of the department before the pandemic. Table 1 summarizes the details of Architectural Drawing and Furniture Design as project-based learning courses.

**Table 1:** Summary information of the architectural drawing and furniture design courses

Course name	Architectural drawing	Furniture design
Course type	Practical engineering drawing course	Practical design course
Course aim	Training students to identify and draw the main architectural elements in architectural drawing	Study of the design methods that highlight the forms of items of simplified furniture
Course topics	The topics of the courses were divided into three main subjects interspersed with sub-exercises to achieve their aims	
	1—Drawing plan, drawing horizontal and vertical architectures 2—Electrical and plumbing architectural drawings 3—Designing architectural facades	1—Drawing furniture with correct dimensions 2—Basics of furniture design by redesigning furniture 3—Creating fresh, new furniture designs
Course traditional approach	Three hours a week in the design studio	Four hours a week in the design studio

### Development of online courses during the early phase of the pandemic

**Fig. 3:**
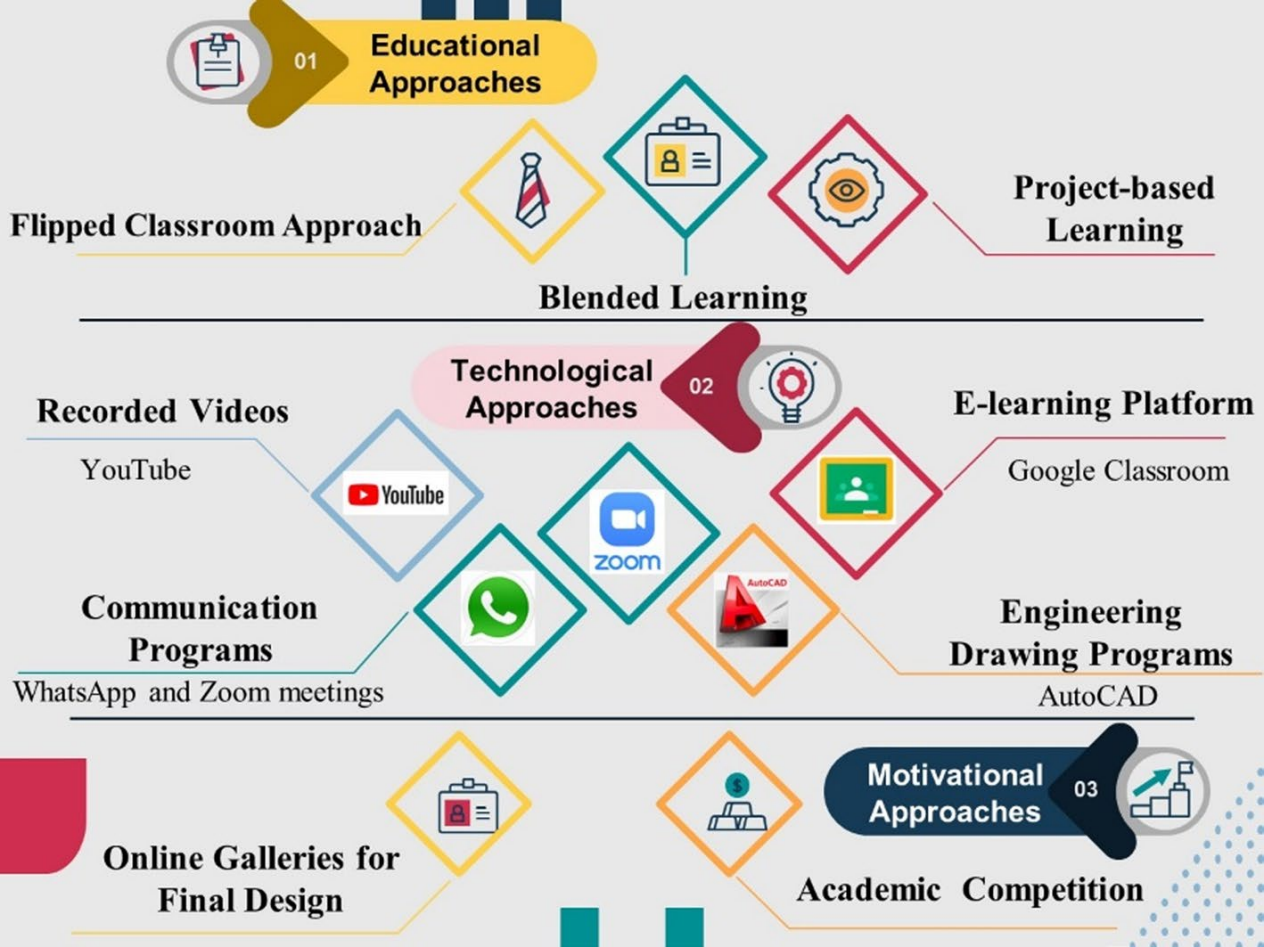
Summary of our methods and approaches in the developed courses. The template design application used is PresentationGO

Students had to adopt distance learning methods at the beginning of the second semester of the academic year 2019–2020 because of the COVID-19 pandemic. The educational approaches that were developed for these practical courses relied on several teaching methods to achieve the best results in achieving each course’s aim. Figure 3 summarizes the methods and approaches we used for developing the courses.

**Fig. 4:**
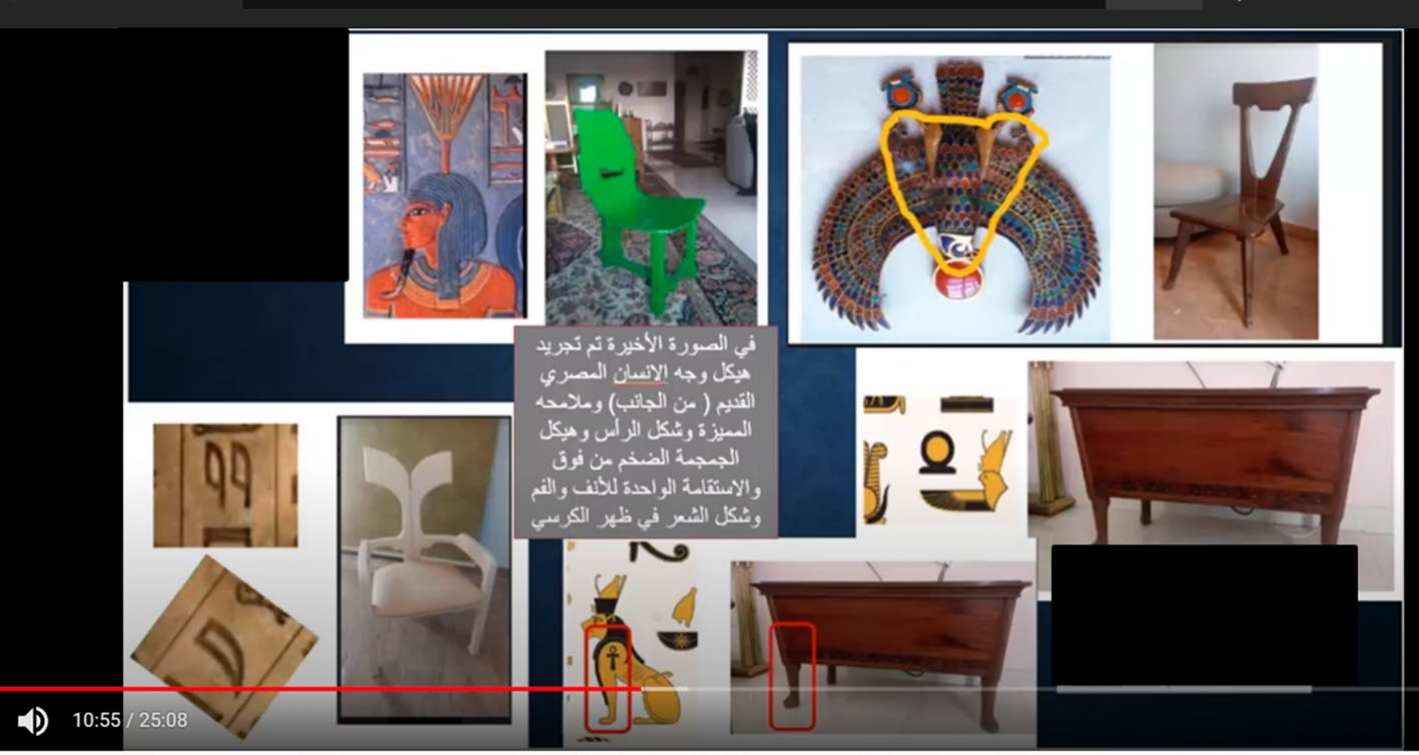
Samples of an inverted Furniture Design session to analyze the furniture design of Professor Ismail Awaad

Many educational approaches were adopted to facilitate the transformation of the traditional teaching model with passive learning pedagogy and engage students in interacting with professors. The flipped classroom model was applied for delivering the two courses to increase student participation and develop critical thinking (Gaida et al., 2016). In the Architectural Drawing course, a discussion was conducted on well-known facade designs. In the Furniture Design course, the designs of Professor Ismail Awaad were presented to the students as part of a task to estimate the original motives of the design as a reverse design process, and all the students engaged successfully. As an alternative to describing the concept behind furniture and its motif design, we exposed the students to something completely different. Professor Ismail Awaad, whose work was significant in restoring the national legacy of Egypt, offered the final designs of his furniture to students, who were encouraged to use their imagination to piece together the inspiration behind Awaad’s motifs and shapes. To gauge the students’ level of achievement in this activity, a discussion was held, during which sections from the designer’s book were presented. Professor Ismail Awaad was shown these findings; he was quite pleased with the students and supported them. Figure 4 presents samples of an inverted Furniture Design session to analyze the Furniture Design pieces of a famous Egyptian furniture designer, Professor Ismail Awaad. https://www.youtube.com/watch?v=p-7pE-BmifA&t=656s

**Fig. 5:**
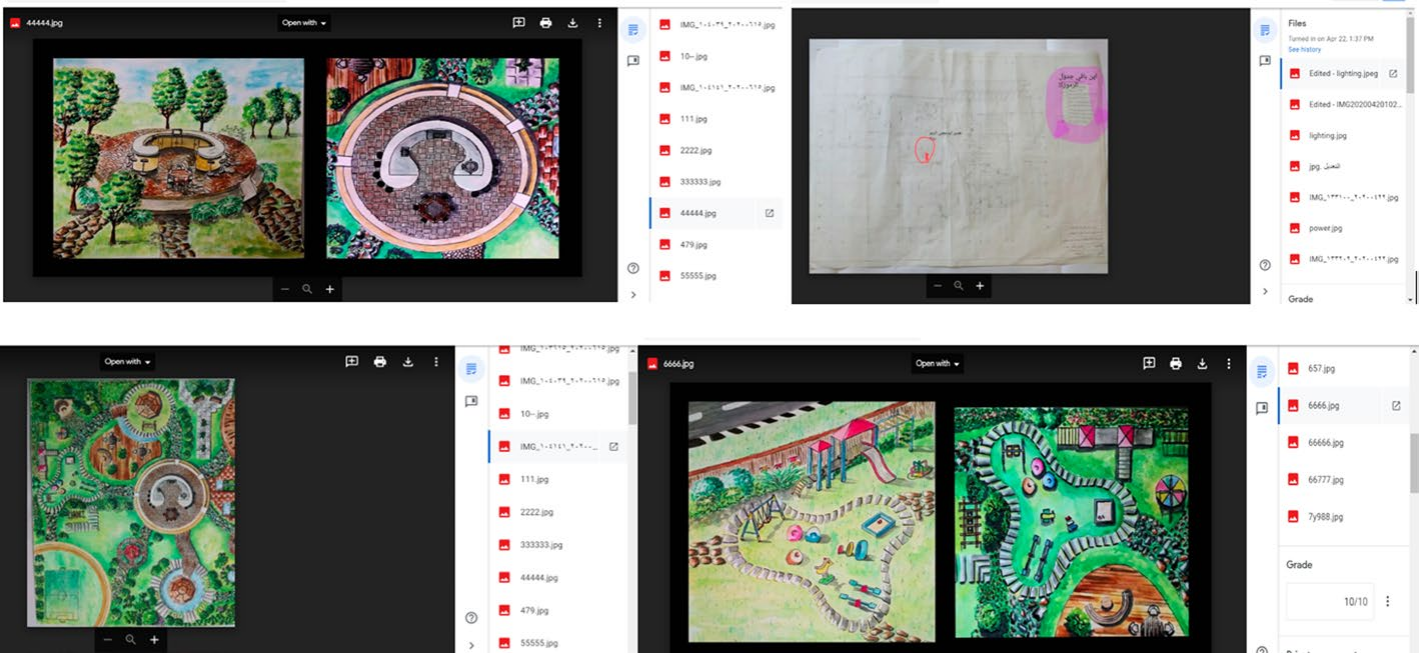
Samples of the online work of the students in the Architectural Drawing course 2019–2020

**Fig. 6:**
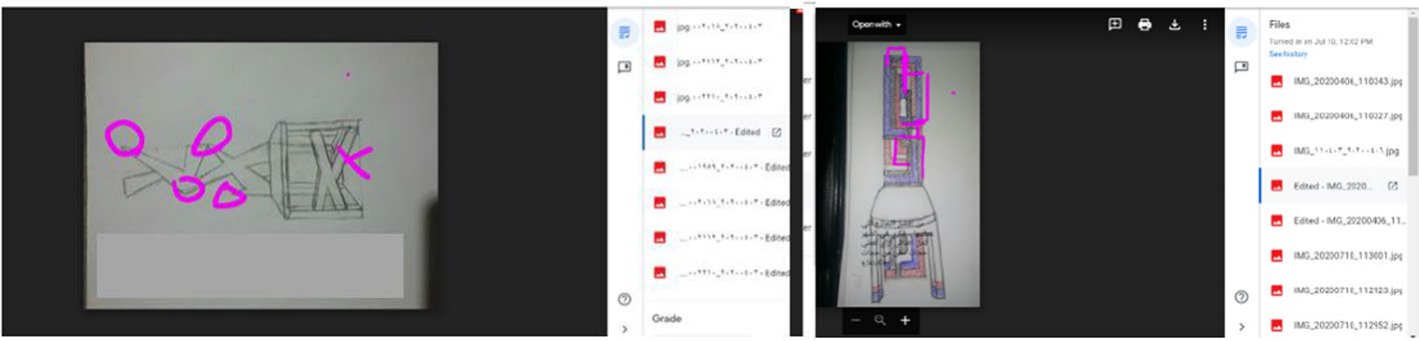
Samples of the online work of the students in the Furniture Design course 2019–2020

**Fig. 7:**
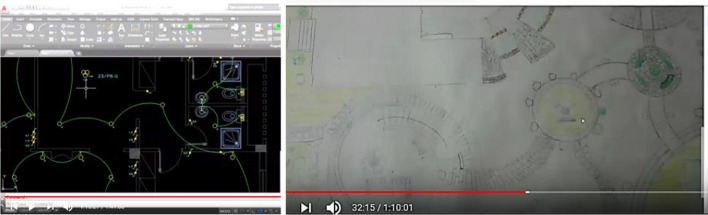
Samples of recorded lectures on YouTube

Blended learning involves combining classroom experience with online activities (Fewella et al., 2021). Technological approaches in design courses must be processed when transitioning to distance learning courses. The following is an explanation of how applications were used in the process:

1. WhatsApp was the best means to quickly and directly interact with students and for setting appointments for simple questions, but it was not entirely successful as an educational platform. WhatsApp was the usual communication channel used by students even before the pandemic, and students requested the creation of a group chat for emergency communications.
2. There was a need for interactive communication and discussion meetings with students, especially in the design course. Although the use of Zoom solved this problem, not all students had access to sufficiently fast internet connections to operate Zoom; therefore, we decided to record the lectures. Ready accessibility to Zoom without a school e-mail account is a significant advantage. Because of the rapid spread of the pandemic, many students could not access their university e-mail accounts as they didn’t need to access it to use it before. The only drawback of using the free version of Zoom was that meetings had to be restarted every 40 min.
3. Google Classroom was used as an educational platform. It is simple and easy to manage, and the content is organized; however, it requires consistent internet connectivity. The essential aspect distinguishing it from other LMSs is that it saves files to Google Drive; thus, it does not consume space on phones and videos are uploaded on YouTube. We shared links on Google Classroom to save space on Google Drive. Figure 5 shows the students’ online work samples that were included in Google Classroom in the Architectural Drawing course 2019–2020. Figure 6 presents the work samples of the students enrolled in the Furniture Design course 2019–2020.
4. YouTube was an ideal open platform for uploading videos, where students could watch them at any time, and it did not consume phone storage space but required an internet connection. Figure 7 presents samples of recorded lectures on YouTube by blending AutoCAD with traditional drawings as an explanatory tool.
5. AutoCAD, an engineering drawing program, is an important tool for clarifying many points of interest in the students’ drawings.

### Addition of online academic competition and an online gallery as a motivational strategy during the pandemic

**Fig. 8:**
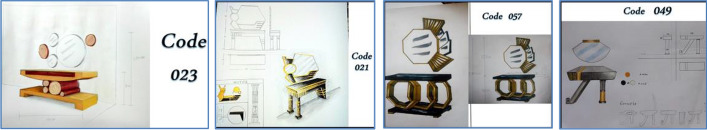
Samples of the online designs of the students in the competition

Many students stopped showing up to class; consequently, their passion and desire for creative work, which had been high before the pandemic, started to dwindle. Thus, we decided to host a Furniture Design competition for first-year students. The contest was based on a course’s available units’ content. With the aim of energizing the student body, the university’s administration and staff supported the competition. Under the department’s watchful eye, a competition named “Challenge COVID-19 and Innovate at Home” was launched in April 2020. Figure 8 shows samples of the students’ online designs in the competition.

Twenty students out of 21 participated in the competition. In the past, students hesitated to participate in competitions because they perceived discrimination when the same persons consistently won first place. Students participated in encoding drawings to protect the designers’ identities, which helped alleviate any feelings of unfairness or favoritism they may have had. This inspired most students to participate in the competition and electronic exhibition. Participation in the competition was not compulsory and not connected to grades; therefore, the students coded their designs for the anonymous survey. It was important for students not to feel more pressure than the depression they felt when the pandemic started. We created an online exhibition for 20 designs and uploaded it to a free website, allowing us to vote for the top five designs.

**Fig. 9:**
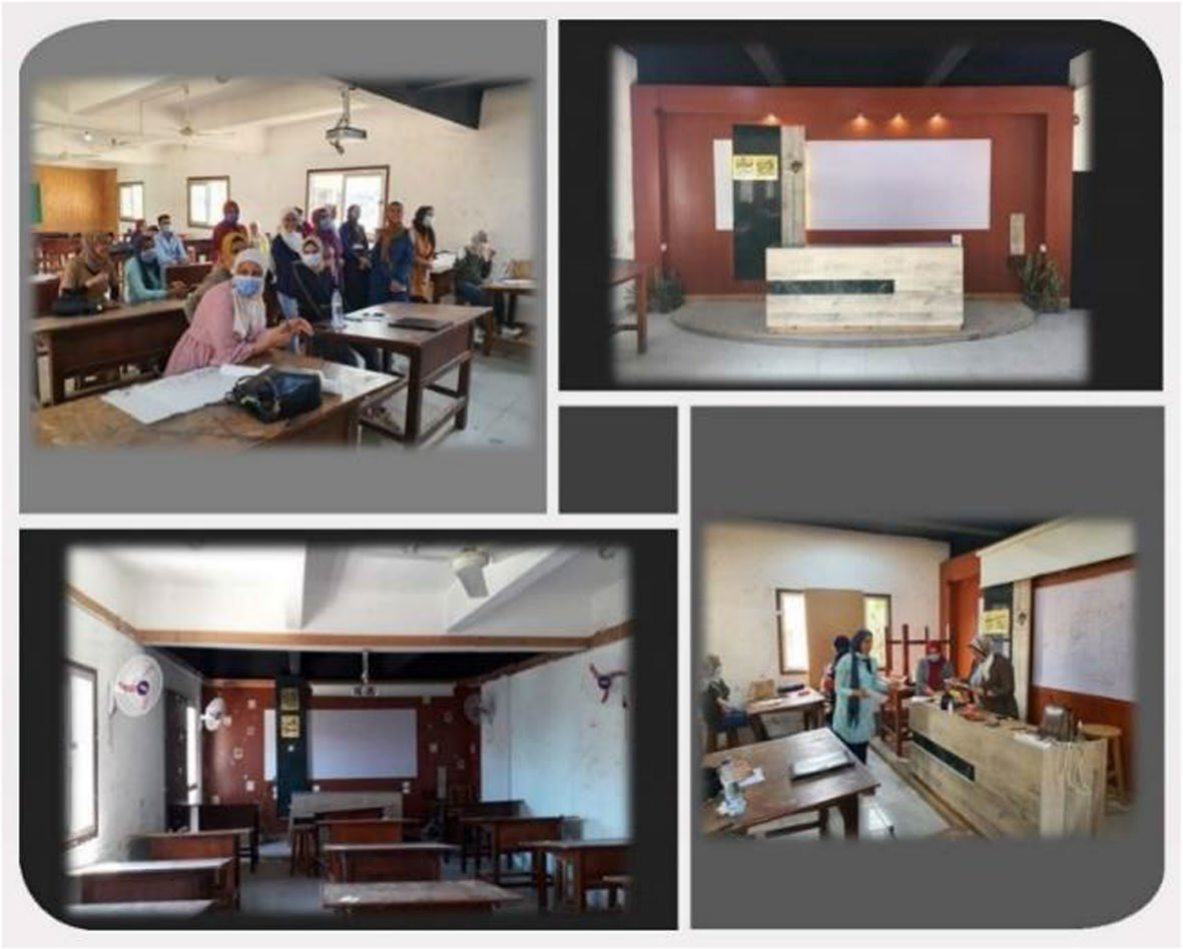
Images of the design studios and the ceremony in honor of the first-year students after the pandemic

We sent the exhibition and voting links to faculty members and furniture specialists. At the end of the specified voting period, an online ceremony was held via Zoom. Figure 9 shows the ceremony that was organized in honor of the first-year students in the design studios after they returned to the faculty in the hybrid learning mode for their efforts during the e-learning period.

**Fig. 10:**
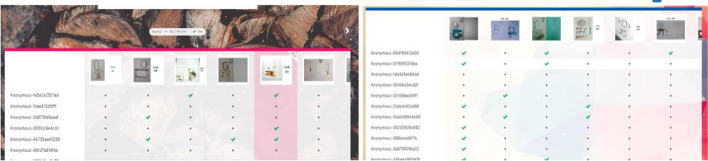
Samples of the online exhibition and voting for the competition

The online voting process ended on 15 April 2021, with 55 specialist votes for the first and second stages. Figure 10 shows the samples of the online exhibition and voting for the competition.

**Fig. 11:**
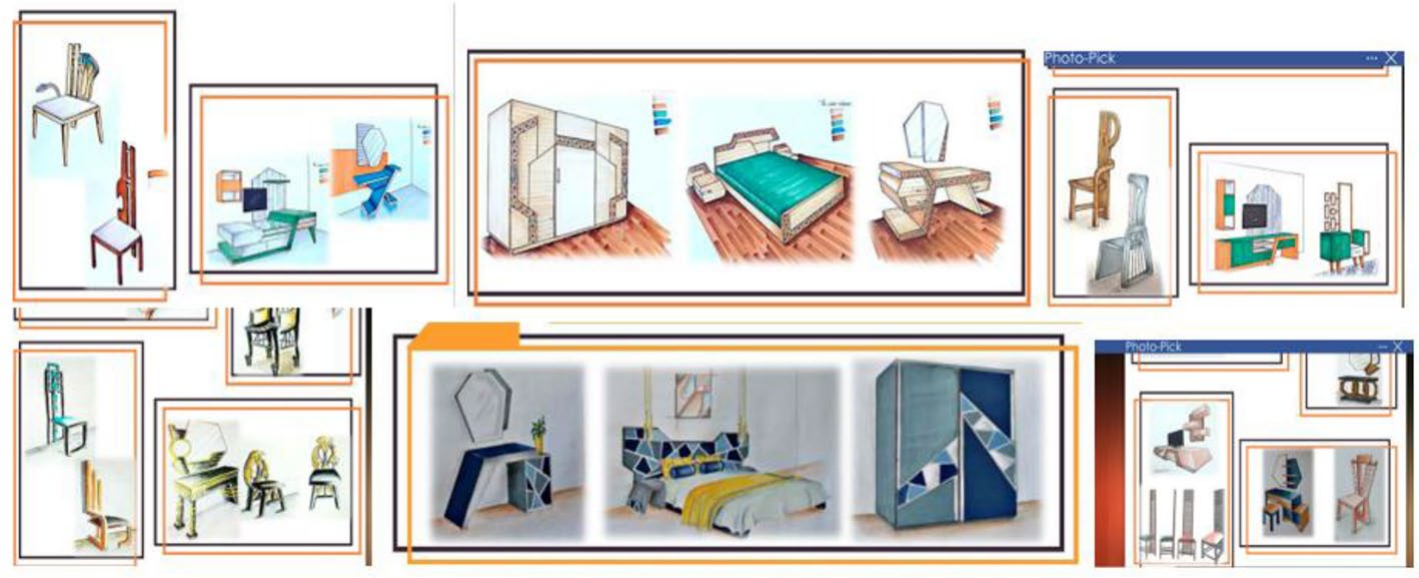
Samples of the final Furniture Design gallery in the course

We developed an online art gallery to motivate students to complete their final artworks at the end of the course. Visitors to the exhibition exceeded 5,000; this was much higher than the numbers expected under normal circumstances. Thus, the pandemic turned from a curse into a blessing as students got the opportunity to broaden the reach of their work. Figure 11 shows samples of the final Furniture Design gallery in the course.

The online competition and exhibition were new to the students and faculty because competitions and exhibitions are traditionally held in gallery halls with considerable time to prepare.

### Evaluation of the online competition and online courses

The students evaluated the impact of e-learning through an online questionnaire using Google Forms with 27 questions. We divided the questionnaire into the following two sections: the effectiveness of online competitions in interior design and furniture and the progress of the courses developed during the pandemic. We also sent the survey to all 21 students enrolled in this course. Seventeen students responded to the first part of the questionnaire about the competition. Table 2 summarizes the online questionnaire design. The online link for the first questionnaire is provided below. https://docs.google.com/forms/d/1F5CYisCQtYLGT238nN4IZ0MZIoWWtsyNpjBMMaKSZZU/edit#responses

**Table 2:** Summary of the first online questionnaire design

Question	Answer type	Proposed answers
1. Did you participate in the “Challenge COVID-19 and Innovate at Home” competition?	Multiple choice	Yes No
2. Do you wish to participate in other competitions of this specialty?		
3. How effective was the idea of coding designs and anonymizing participants?	Multiple choice	Excellent Very well Good Weak Very weak
4. The best five pieces in the group were selected by specialists in the field and faculty members from different universities. What is your assessment of the selected judges?	Multiple choice	Academic staff and specialists Public
5. What is your assessment of the method the judges used in selecting the winners?	Multiple choice	The arbiters chose the best works by working with an open online gallery voting system They created specific criteria for choosing the winners They made a points table, and the designs with the highest score were chosen
6. What is your assessment of the idea to hold a competition and its effect on motivation during distance learning?	Multiple choice	Excellent Very well Good Weak Very weak
7. Do you think there should be stricter criteria for accepting entries for the next competition?	Multiple choice	Yes No Maybe
8. Do you support the establishment of competitions for each class separately or all the students in the department?	Multiple choice	Each class separately The entire department
9. Did winning or losing in the competition motivate you to continue to be creative?	Multiple choice	Yes No Maybe
10. Determine your proposals for the style of academic competitions	Short answer	–

https://docs.google.com/forms/d/1F5CYisCQtYLGT238nN4IZ0MZIoWWtsyNpjBMMaKSZZU/edit#responses

Twenty students responded to the second part about the developed courses. Table 3 summarizes the second online questionnaire design. The online link for the second questionnaire is presented below. https://docs.google.com/forms/d/1EfEmyVLKD-W_zYsVCMA0yauTCZLYP2Eyjl8wfXwrlW0/viewanalytics

**Table 3:** Summary of the second online questionnaire design

Question	Answer Type	Proposed Answers
1. At the end of the second semester in the Furniture Design and Architectural Drawing courses, to what degree do you believe you attained your objective for undertaking the courses?	Multiple choice	Excellent Very well Good Weak Very weak
2. How do you evaluate the scientific content of the courses?		
3. During the COVID-19 pandemic this semester, how do you evaluate the performance of the lecturer during distance learning?		
4. How do you evaluate the relevance of the course content in terms of information related to your specialization?		
5. How do you assess your understanding of the course content through distance learning after the COVID-19 pandemic?		
6. If the conditions of the COVID-19 pandemic continue, can the content of these materials be provided online?	Multiple choice	Yes, architectural drawing and furniture design content can be provided online Yes, but only architectural drawing content can be provided online Yes, but only furniture design content can be provided online The content of the two courses cannot be taught through distance learning
7. What is your assessment of the method of accessing the materials provided for the two courses?	Multiple choice	The content is easy to access The content is challenging to access I experienced difficulties in accessing the course materials
8. To what extent has an interactive program such as Zoom helped you?	Multiple choice	It helped me very much It helped slightly It did not help me
9. What system of lecture presentation do you prefer in the distance learning system?	Multiple choice	Video lectures on YouTube Interactive lectures on an online program such as Zoom PDF file Google Classroom discussions
10. To what extent are you satisfied with the evaluation system on the Google Classroom program?	Multiple choice	Very satisfied Slightly satisfied Not satisfied
11. What are your most significant difficulties in using the distance education system?	Multiple choice	Internet connectivity issues Lack of availability of devices Difficulty in communicating with the lecturer All of the above Others
12. To what extent did the launch of the “Challenge COVID-19 and Innovate at Home” competition motivate you to work?	Multiple choice	It helped me a lot It helped me a little It did not help me
13. How often did the work in both courses run on a system during distance learning using the Zoom, Google Classroom, and WhatsApp programs?	Multiple choice	Very regularly Slightly regularly Irregularly
14. To what extent do you consider the importance of distance learning in maintaining student health as a temporary alternative to traditional education?	Multiple choice	Very necessary It can help It does not help and should not be used instead of traditional education
15. What are your suggestions for developing and teaching the architectural drawing course?	Short answer	–
16. What are your suggestions for developing and teaching the furniture design course?	Short answer	–
17. What are your suggestions for developing the distance education system considering the COVID-19 pandemic?	Short answer	–

## Results

Procedures were carried out, including an evaluation of students’ reactions to the competition to assess the outcomes of this learning experience. After the course, students’ final design projects were shown digitally, and they filled out a questionnaire about their overall impressions of the learning experience.

### First part of the questionnaire regarding the online competitions

**Fig. 12:**
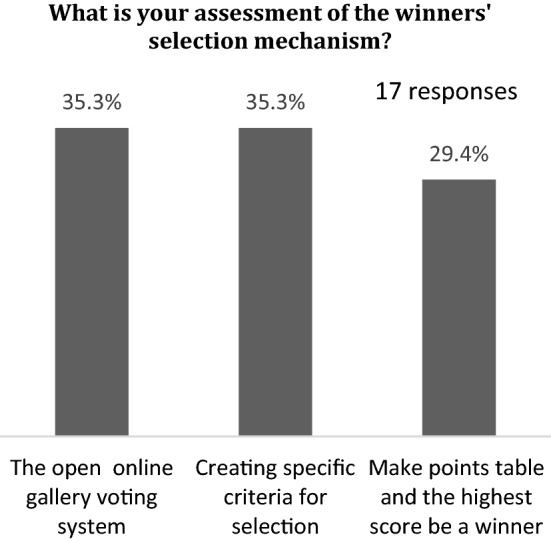
Student preferences of the judgment of the prize-winning works

**Fig. 13:**
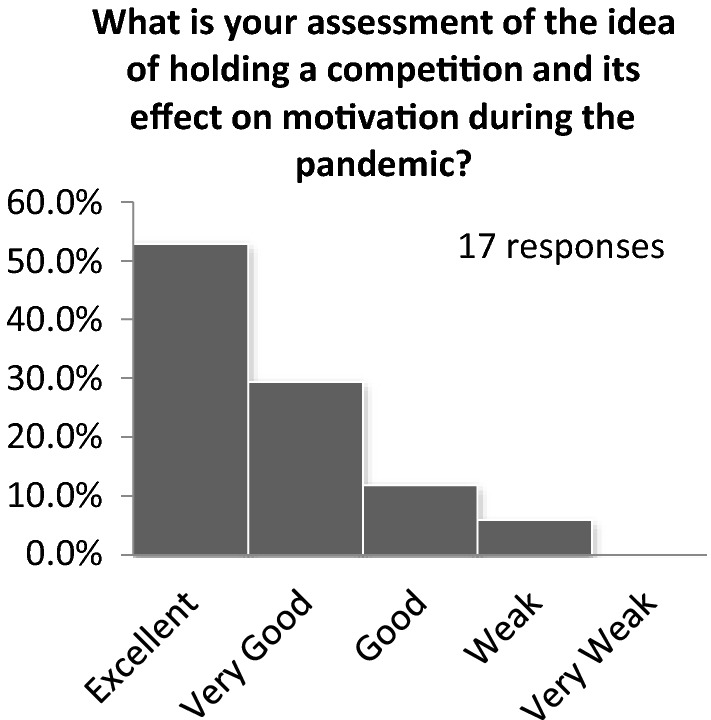
Student evaluation of the competition during the pandemic

The first part of the questionnaire was about students’ evaluation of the online competition’s effects. Almost 82% of students wished to participate again in competitions. Approximately 70% found that the idea of coding designs to anonymize designers was excellent. The students had been exposed to contests where experts shortlisted the candidates, and then the public voted for the top contenders. In other words, if two people are equally qualified, the one with more friends will ultimately prevail. Therefore, the students voted to keep the design creator’s identity a secret and limit the voting to experts only. Nearly 94% chose to limit the selection of competition judges to university professors and field specialists. Figure 12 displays the student preferences for the selection criteria. The open online gallery voting system and the creation of specific criteria for selection were highly preferred. Figure 13 shows the student responses to the evaluation of the competition’s effectiveness on motivation during the pandemic; almost 53% found that it was excellent.

Most students (88%) supported the establishment of the competition for each study class separately. Almost 76.5% of respondents agreed that regardless of winning or losing, the competition had inspired them not to lose their passion.

### Second part of the questionnaire regarding the courses developed during the pandemic

**Fig. 14:**
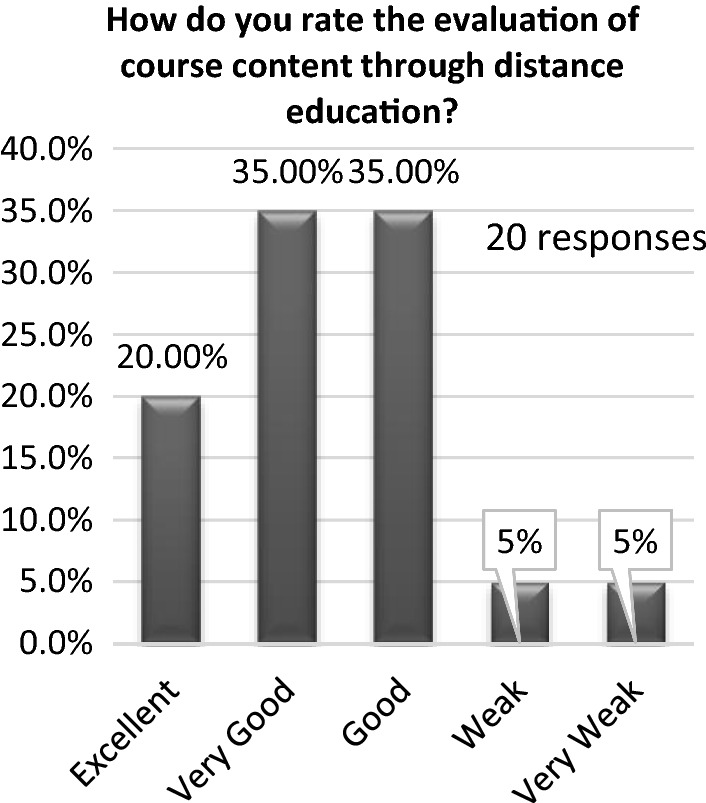
Evaluation of students’ understanding of the distance learning course content

**Fig. 15:**
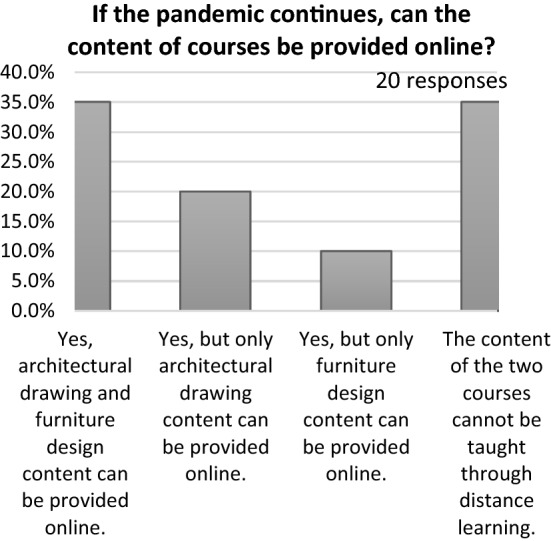
Opinions of students regarding the courses they would like to undertake online if the pandemic continues

The second questionnaire evaluated the courses developed during the pandemic. Half of the students found that the content was good, while the other half found it to be excellent. Additionally, almost 65% affirmed that the lecturer’s performance during the pandemic was excellent. Figure 14 exhibits the evaluation of the students’ understanding of the distance learning course content. One-third (35%) found that the content was very good, and approximately 35% found that it was good. Figure 15 illustrates students’ opinion regarding the courses they would like to undertake online if the pandemic continued. The students could not confirm this because 35% found that the course could be provided online, and the same percentage found that it could not be delivered online.

**Fig. 16:**
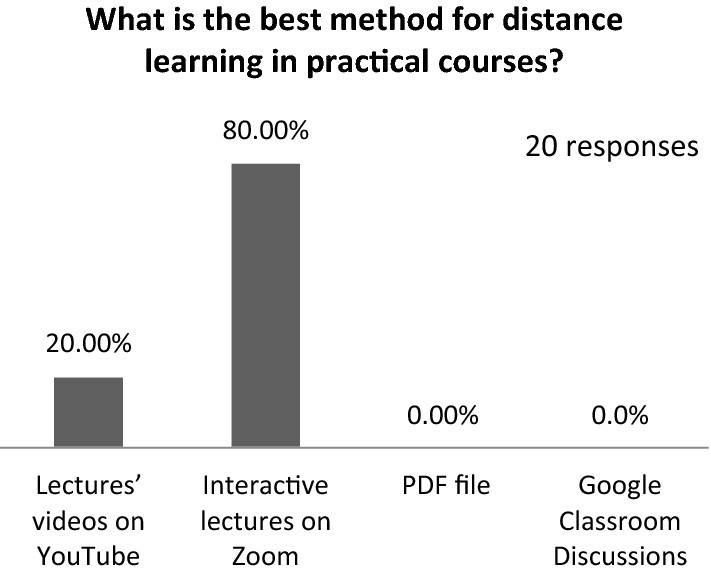
Student preferences for distance learning in practical courses

**Fig. 17:**
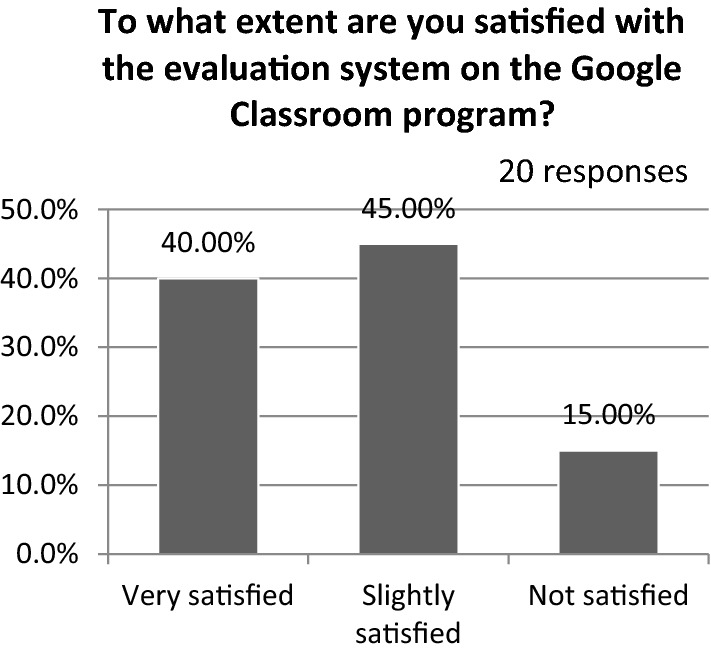
Student satisfaction with Google Classroom’s evaluation system

The majority (90%) found the content easy to access, and almost 80% asserted that using an interactive program, such as Zoom, helped them substantially. Figure 16 shows the student preferences for distance learning in practical courses, and almost 80% confirmed that they preferred interactive lectures through platforms such as Zoom. Figure 17 presents student satisfaction with Google Classroom’s evaluation system; nearly 45% affirmed that they were slightly satisfied.

Almost 85% students asserted that they had internet connection problems while they attended distance education classes. Roughly 70% disclosed that the competition motivated them to work. The students suggested in the course development that they should have pre-course drawing programs. Other students hoped to organize field visits to sites. Some students noted that the university should provide them with free internet connection for e-learning.

### Findings and analyses

The study was conducted in Egypt, and online education in the art sector was not widespread before the pandemic. Distance learning is still in its early stage in developing countries. Educational systems and scientific materials need to be developed further. Ceylan et al. (2021) found that hybrid techniques for design education are best for the design studio. In 2020, people’s understanding of these techniques was enhanced, and schools were prompted to consider them when creating design studio frameworks because of the pandemic conditions. Not all architectural schools in Egypt are prepared for e-learning, and most have never used this learning mode. The psychological effects of working from home have affected students. However, for the successful implementation of e-learning, internet issues must be resolved, staff and students must be taught to utilize online tools for virtual classrooms, and solid e-learning platforms should be created. The future of traditional studios is unclear (Elrawy & Abouelmagd, 2021). Many students have had trouble keeping up with online coursework. In some cases, people’s only access to the web is through their mobile devices because they reside in remote areas or rural villages. The high cost of mobile internet access makes it impractical to use for virtual conferences. Figure 14 shows that this stratification resulted in two distinct groups of students: those who thrived on the ongoing dialogue of that phase and preferred to repeat it and those who struggled to find any utility in it.

The research presents the experience of applying distance learning for the first time in practical courses in the COVID-19 pandemic’s context to develop a general strategy that can be applied in future scenarios. The developed courses were the first to be taught out of the design studio in the university. The student number in the courses was relatively small because of the low number of students accepted in the department based on the seats available in the classrooms. Therefore, future studies should be conducted with more students.

The results confirmed that students were moderately satisfied with the course content; hence, the content should be developed further, and specialized multimedia could be added. Students also preferred interactive programs, such as Zoom, for lectures. Distance learning platforms must be developed to attract students, considering the ease of use and access to content. Providing university support for internet access is preferable considering the internet connectivity issues faced by a large percentage of students. Furthermore, training students in engineering drawing programs early in the specialization is favorable to enable them to efficiently complete their tasks because first-year students were not yet trained in engineering drawing programs, which made it challenging to perform the required tasks without manual tools.

During the quarantine period, students had trouble getting access to the stationary items and specialized engineering tools they needed to do their tasks. The instructor observed the students’ sketching attempts on recycled paper and assumed they would be imaginative. However, these issues could be mitigated if the learners are trained to use engineering drawing software. Their papers, hand drawings, and filming videos could be of poor quality, particularly in the Architectural Drawing course, which requires high precision in drawings, so instructors were allowed to utilize engineering programs as an explanation technique. The students in these webinars utilized Zoom to discuss and modify their designs for the Furniture Design course, which they drew by hand and had the professor correct in real-time using Google Classroom and Zoom.

**Fig. 18:**
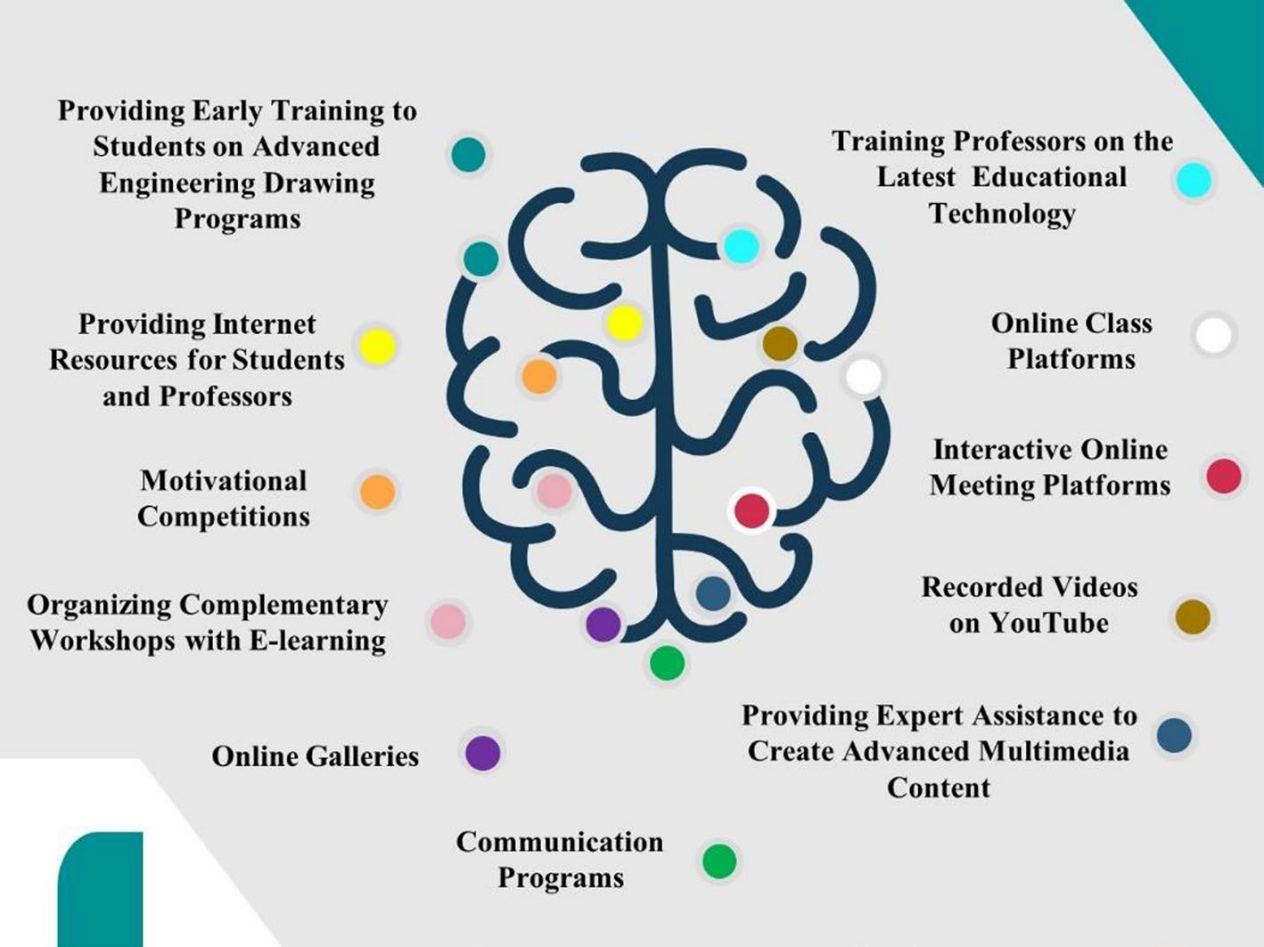
The general strategy for the development of distance education in arts and engineering. The template design application used is PresentationGO

The students explained that academic competitions and electronic exhibitions motivated them to work under the conditions created by the pandemic. Figure 18 presents the final general strategy for the development of distance education in the fields of arts and engineering. Also, Fallatah’s (2021) study on senior interior design students’ perspectives of distant learning during COVID-19 suggested the following: (* (a) Hybrid teaching technique (b) One-on-one teacher meetings (in person) (c) Finals on campus (d) Additional tutorials for practical course tasks (e) More exams and fewer projects in theoretical courses (f) More project.)

## Conclusions and recommendations

The COVID-19 pandemic has had an immense impact on higher education, especially practical faculties dependent on project learning in design studios and in-person work. The Architectural Drawing and Furniture Design courses were chosen as study models in this research. These courses had been taught only through traditional approaches using paper, pencil, and ruler. Today, digital technologies and related applications are increasingly being used to teach drawing courses. Connection to the internet and e-learning platforms enable the teachers and learners to access and use information stored online. Thus, e-learning complements classroom lessons. During the courses, the students could view all online resources at any time, allowing them to review the subjects discussed during the lecture regularly. The e-learning program also complements the lecturer’s lessons. Establishing electronic competitions and online exhibitions motivates students to finish their work in the context of online education.

This paper fills the gap between classic higher education systems and e-learning in practical faculties. This gap is not restricted to facilitating regular meetings with professors; students can also participate in these meetings. This research presents students’ experiences of e-learning in practical faculties in Egypt, a developing country. Educational institutions should develop programs to attract both domestic and international students based on the results. The study can be beneficial in helping decision-makers in higher education sectors of developing countries recognize students’ perspectives.

Furthermore, universities must focus on approaches and policies that will enhance students’ acceptance of e-learning. Various approaches must also be undertaken, such as creating high-quality content, offering hands-on skill-enhancing opportunities, and organizing live and interactive workshops or question-and-answer sessions. These include verifying the authenticity of students completing the course and the stringent monitoring of online course providers to eliminate fake courses. Consistently updating online courses will surely promote the adoption of e-learning (Phutela & Dwivedi, 2020). Student evaluation analysis revealed a few subtle issues leading to greater student progress and assistance for further framework improvements.

## Limitations and implications for further research

Considering the need for a dedicated space for each student, a drawing table, and other equipment, small numbers of students are accepted to the Department of Interior Design and Furniture. The small sample size (n = 21) of the present study was small; therefore, more testing should be conducted on a broader population. Because of the various challenges presented by the study’s location at a regional university in a developing country, it is recommended that future research be conducted in universities with more resources. The research was done at the beginning of the COVID-19 pandemic. It could be useful to conduct this research again after students and instructors have more background knowledge regarding e-learning and access to modern technological resources.
